# Impact of Combining Long-Term Subsoiling and Organic Fertilizer on Soil Microbial Biomass Carbon and Nitrogen, Soil Enzyme Activity, and Water Use of Winter Wheat

**DOI:** 10.3389/fpls.2021.788651

**Published:** 2022-02-08

**Authors:** Yonghui Yang, Minjie Li, Jicheng Wu, Xiaoying Pan, Cuimin Gao, Darrell W. S. Tang

**Affiliations:** ^1^Institute of Plant Nutrition and Resource Environment, Henan Academy of Agricultural Sciences, Zhengzhou, China; ^2^Yuanyang Experimental Station of Crop Water Use, Ministry of Agriculture, Yuanyang, China; ^3^Field Scientific Observation and Research Station of Water Saving Agriculture in the Yellow River Basin of Henan Province, Yuanyang, China; ^4^Department of Bioengineering, School of Life Sciences, Zhengzhou University, Zhengzhou, China; ^5^Soil Physics and Land Management Group, Wageningen University and Research, Wageningen, Netherlands

**Keywords:** subsoiling, organic fertilizer, combined amendments, soil microbial biomass carbon and nitrogen, water use, yield

## Abstract

Reductions in soil productivity and soil water retention capacity, and water scarcity during crop growth, may occur due to long-term suboptimal tillage and fertilization practices. Therefore, the application of appropriate tillage (subsoiling) and fertilization (organic fertilizer) practices is important for improving soil structure, water conservation and soil productivity. We hypothesize that subsoiling tillage combined with organic fertilizer has a better effect than subsoiling or organic fertilizer alone. A field experiment in Henan, China, has been conducted since 2011 to explore the effects of subsoiling and organic fertilizer, in combination, on winter wheat (*Triticum aestivum* L.) farming. We studied the effects of conventional tillage (CT), subsoiling (S), organic fertilizer (OF), and organic fertilizer combined with subsoiling (S+OF) treatments on dry matter accumulation (DM), water consumption (ET), water use efficiency (WUE) at different growth stages, yield, and water production efficiency (WPE) of winter wheat over 3 years (2016–2017, 2017–2018, 2018–2019). We also analyzed the soil structure, soil organic carbon, soil microbial biomass carbon and nitrogen, and soil enzymes in 2019. The results indicate that compared with CT, the S, OF and S+OF treatments increased the proportion of >0.25 mm aggregates, and S+OF especially led to increased soil organic carbon, soil microbial biomass carbon and nitrogen, soil enzyme activity (sucrase, cellulose, and urease). S+OF treatment was most effective in reducing ET, and increasing DM and WUE during the entire growth period of wheat. S+OF treatment also increased the total dry matter accumulation (Total DM) and total water use efficiency (total WUE) by 18.6–32.0% and 36.6–42.7%, respectively, during these 3 years. Wheat yield and WPE under S+OF treatment increased by 11.6–28.6% and 26.8–43.6%, respectively, in these 3 years. Therefore, S+OF in combination was found to be superior to S or OF alone, which in turn yielded better results than the CT.

## Introduction

Water scarcity and seasonal drought are major constraints on agricultural development globally. Henan province, the main wheat producing region of China, accounts for a quarter of total wheat production in the country (Wu et al., 2003). In addition, due to historical rotary tillage practices, the soil structure has become suboptimal for agriculture: evaporation rates have increased and the soil moisture retention capacity has decreased (Hemmat and Eskandari, 2004; Vita et al., 2007), which further exacerbates the imbalance between water supply and demand. Thus, wheat yields in the area are severely limited by water deficiency. Therefore, improving the utilization efficiency of limited water resources and alleviating the damage caused by drought stress to crops has become an important issue (Li et al., 2015).

Effective tillage and fertilization measures can improve soil properties, increase the water use efficiency of the crop, and increase yields (Sang et al., 2016; Wang et al., 2016; Bottinelli et al., 2017; Zhang et al., 2017). Subsoiling can break the bottom of the plow layer, deepen the soil tillage layer, improve soil pore characteristics, enhance soil infiltration and moisture retention capacity, and increase water use efficiency (Zheng et al., 2011; Sang et al., 2014) and crop yields (Mohanty et al., 2007; Yan et al., 2011; Hu et al., 2013; Yang et al., 2016; Li et al., 2019). Studies have shown that subsoiling can maintain higher physiological activity in flag leaves, increase the accumulation of dry matter in the middle and later stages of wheat growth, and delay the senescence of wheat plants (Zhang et al., 2008; Chu et al., 2012). In addition, Piovanelli et al. (2006) and Kuzyakov and Xu (2013) found that subsoiling can improve crop root growth, which helps maintain optimal plant growth, increases the activity of urease and sucrase in the soil (Sun et al., 2019), while also increasing root stubble and root secretions, which in turn increase the growth and capacity of microorganisms in the soil, thereby activating soil nutrients and promoting nutrient absorption by crops.

The application of organic fertilizers can also improve soil fertility and soil structure, and increase soil water storage and retention capacity, which then improves photosynthetic capacity, water use efficiency and crop yield (Lu et al., 2011; Karami et al., 2012; Liu et al., 2013). Wang et al. (2020) found that organic fertilizers increased grain yield and water use efficiency by an average of 18 and 20% compared to inorganic fertilizer. Studies have also shown that organic fertilizer can improve photosynthetic capacity and delay leaf senescence, which beneficial for increasing the accumulation of dry matter above ground (Dordas, 2009; Bogard et al., 2010). Furthermore, organic fertilizers are a source of carbon, nitrogen, and microorganisms, which provides energy and material for the growth of microorganisms in the soil, and increases the microbial composition of carbon and nitrogen in the soil (Elfstrand et al., 2007). Liang et al. (2010) showed that compared with non-fertilization, the long-term application of organic fertilizer can increase microbial carbon and nitrogen content in the soil by 1.4–2.7 times and 1.9–2.5 times, respectively. However, Zhang et al. (2013) found that single application of organic fertilizer had no significant impact on microbial carbon volume in the soil. Some studies (Bu et al., 2006; Rehana et al., 2008) have shown that long-term application of organic fertilizer can improve the activity of different types of enzymes in the soil, especially urease, sucrase and protease (Pu et al., 2020).

As discussed above, most prior studies have focused on the effects of solely subsoiling or organic fertilizer treatment. Few studies have systematically studied the impact of long-term application of these treatments and the combined application of subsoiling and organic fertilizer, on soil structure, soil microbial biomass carbon and nitrogen, soil enzyme activity, soil water and water use of crops, and any possible correlations between these outcomes. We hypothesize that subsoiling combined with organic fertilizer has a better effect on physical and chemical properties and water use of wheat than subsoiling or organic fertilizer alone, in alleviating or resolving crop damage due to seasonal drought and water scarcity. This study is based on 3 years of results from a long-term experiment, and studies the influence of the individual practices and their combined application on crop water production efficiency (WPE) and yield. We aim to also investigate the mechanisms underlying the effects of these practices, to provide a theoretical basis that would lead to efficient crop water use and yield increases in the region and other geographically similar regions in China and elsewhere. To accomplish this, we analyze the effects of these treatments on soil microbial biomass carbon and nitrogen, soil enzyme activity, dry matter accumulation, and water use efficiency at various growth stages of winter wheat, and perform correlation analyses on these experimental outcomes.

## Materials and Methods

### Experiment Location

**FIGURE 1:**
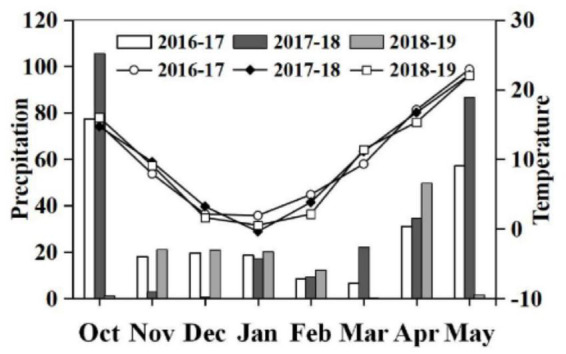
Precipitation and temperature during the growing period of wheat in three seasons (2016–2017, 2017–2018, and 2018–2019).

The experiments analyzed in this study were conducted at the Tongxu Experimental Station (144°26′58.47″E; 34°25′44. 26″ N, 62 m above sea level) of the Water Saving Agricultural Base in the east of Henan province from 2016 to 2019, as part of a long-term experiment started in October 2011. The annual average temperature is 14.2°C. The mean annual precipitation is 675.9 mm, of which approximately 60% is received from July to September. The region is topographically flat with uniform fertility. According to the international texture classification system, the soil type is mostly sand and loam [59.1% sand (2–0.02 mm), 22.5% silt (0.02–0.002 mm), with 18.4% clay (<0.002 mm)] derived from loess soils. At the start of the present experiment, the organic matter, total nitrogen, nitrate nitrogen, ammonium nitrogen, available phosphorus, available potassium, and bulk density in top layer of soil (0–20 cm) were 11.4 g kg^–1^, 0.81 g kg^–1^, 74.31 mg kg^–1^, 55.89 mg kg^–1^, 19.8 mg kg^–1^, 90.3 mg kg^–1^, and 1.3 g cm^–1^, respectively. Crop rotation between wheat and maize has been employed in the area for over 50 years. Figure 1 shows the precipitation and atmospheric temperature during the wheat growing seasons for the years of 2016–2019, and illustrates the rainy growth seasons of 2016–2017 and 2017–2018, and the dry growth season of 2018–2019.

### Experimental Design

In the experiment, a randomized split-plot design was adopted, divided into 12 plots with three replicates: CT (conventional tillage, 15 cm deep, plowing with a rotavator), S (subsoiling, 30 cm deep, loosening the plow bottom layer and the core soil layer with a deep loosening machine without turning the soil layer), OF (organic fertilizer, 15 cm deep, plowing with a rotavator), S+OF (organic fertilizer combined with subsoiling, 30 cm deep, loosening the plow bottom layer and the core soil layer with a deep loosening machine without turning the soil layer). The nitrogen, phosphorus and potassium contents of the organic fertilizer were, respectively, 1.5, 1.2, 0.8%. The plot size was 33.6 m^2^ (5.6 m × 6 m) with wheat spaced 20 cm apart sown at 195 kg hm^–2^. Urea (N, 225 kg hm^–2^), calcium superphosphate (P, 105 kg hm^–2^) and potassium chloride (K, 75 kg hm^–2^), and the total amount of N, P, K applied to all treatments were identical. 50% of the nitrogen fertilizer was applied before sowing, and the remaining 30 and 20% were applied at the jointing and booting stage, respectively. Irrigation was carried out at the jointing stage and grouting stage, respectively, with the amount of irrigation being 60 mm. The wheat cultivars were Bainong 207 in 2016–2017, and Zhengmai 369 in 2017–2018 and 2018–2019. Wheat was sown in the middle of October and harvested in early June.

### Sampling and Measurements

#### Measurement of Soil Water Storage

Soil samples in the soil profile (0–100 cm layer) were collected using soil augers at the sowing, booting, anthesis and harvest stages of wheat, while soil water storage was measured with the oven drying method.

Equations (1) and (2) are used to calculate the soil quality water content and soil water storage (Zhang et al., 2019).


(1) $$SWC\left(\%\right)=\frac{W1-W2}{W2}\times 100$$

(2) $$soil\;water\;storage(\mathrm{mm})=\text{Σ}SWC_{i}\cdot D_{i}\cdot H_{i}\;i(1,\;n)$$

where *SWC* represents the soil water content (%), *W1*(g) represents wet soil weight, *W2* (g) represents dried soil weight, *D*_*i*_ (g cm^–3^) is the soil bulk density of layer *i*, and *H*_*i*_ (mm) represents the soil depth. The water consumption at each growth stage was calculated with Equations (3) and (4) (Guo et al., 2015; Wang et al., 2019).


(3) $$\Delta S=10\text{Σ}(SWC_{il}-SWC_{i2})\cdot D_{i}\cdot H_{i}$$

(4) $$ET_{1-2}=10\overset{\text{n}}{\underset{i=1}{\text{Σ}}}(SWC_{\text{il}}-SWC_{\text{i}2})D_{\text{i}}H_{\text{i}}+I+P\;i(\text{1},n)$$

where *SWC*_*il*_ and *SWC*_*i2*_ represent the soil water content at the bottom of layer *i*, and the soil moisture at the top of layer *i*, respectively; *D*_*i*_ (g cm^–3^) is the soil bulk density of layer *i*, *H*_*i*_ (mm) represents the soil depth; *ET_1–2_* (mm) is water consumption amounts during a growth stage (mm), and *I* and *P* represent the irrigation (mm) and precipitation during the growth period of wheat, respectively.

#### Dry Matter Accumulation and Water Use Efficiency

The 1 m double-rows of wheat plant samples were collected at the jointing stage, booting stage, anthesis stage and harvesting stage, then dry matter was measured with the oven drying method at 70°C. After calculating the dry matter accumulation per unit area, the water use efficiency (WUE) was computed as:


(5) WUE=DM/ET

where *WUE* and *DM* are the water use efficiency and dry matter accumulation of wheat.

#### Grain Yield and Components

At the harvest stage, the number of ears per 0.5 m^2^ in each plot was calculated; wheat plants from a randomly chosen 4 m^2^ area in each plot were harvested and then threshed, air-dried and weighted to calculate the grain yield, and converted into grain yield per unit area. In addition, 10 wheat plants were randomly selected from each plot, their grain numbers were counted, and the grain numbers per ear were calculated. The yield WPE is:


(6) WPE=Y/ET

where *WPE* is yield WPE; *Y* is the grain yield; *ET* is the total water consumption during the entire growth period of wheat.

#### Determination of Soil Microbial Biomass Carbon and Nitrogen, Soil Enzyme Activity, Soil Organic Carbon, and Soil Aggregates

Determination of soil microbial biomass carbon and soil microbial biomass nitrogen was determined with the chloroform fumigation extraction method (Vance et al., 1987; Christianson, 1988; Moore et al., 2000). 3,5-dinitro salicylic acid was used to determine soil sucrase and cellulase activity (Guan, 1986; Qin et al., 2020), while soil urease activity was determined with the indigo phenol ratio method (Wang et al., 2009; Qin et al., 2020), and protease activity was determined with the ninhydrin contrast color method (Guan, 1986; Qin et al., 2020). The soil total organic carbon content was determined using a heavy cadmium acid potassium outside heating method (Westerman, 1990). The size distribution of water-stable aggregates was determined using the wet sieving method (Elliot, 1986). The aggregated soil was separated into different size fractions by gently shaking the samples into the water through a range of sieves to obtain the aggregate size fractions <0.25, 0.25∼0.5, 0.5∼1.0, 1.0∼2.0, 2.0∼3.0, 3.0∼5.0, and >5 mm.

## Statistical Analysis

**FIGURE 2:**
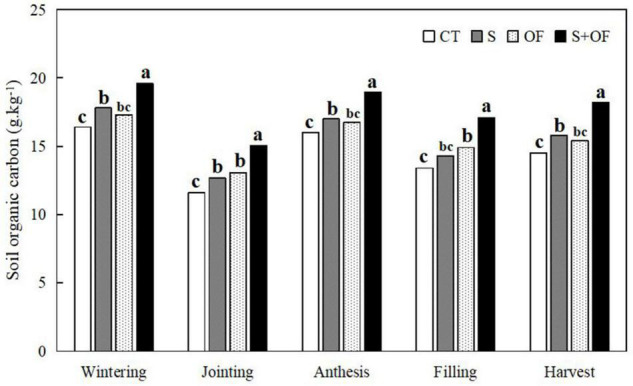
Effects of subsoiling, organic fertilizer and subsoiling combined with organic fertilizer on soil organic carbon at different growth stages of winter wheat in 2019. CT, conventional tillage; S, subsoiling; OF, organic fertilizer; S+OF, organic fertilizer combined with subsoiling. Different lowercase letters in the same growth stage indicate significant differences among treatments by LSD test (*P* < 0.05).

**FIGURE 3:**
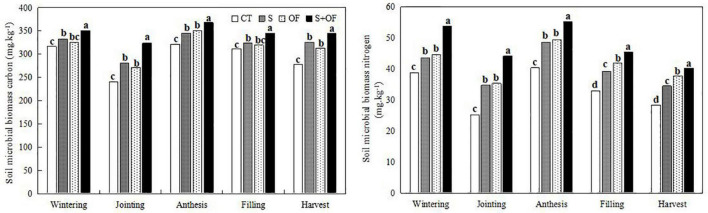
Effects of subsoiling, organic fertilizer and subsoiling combined with organic fertilizer on soil microbial biomass carbon and soil microbial biomass nitrogen at different growth stages of winter wheat in 2019. CT, conventional tillage; S, subsoiling; OF, organic fertilizer; S+OF, organic fertilizer combined with subsoiling. Different lowercase letters in the same growth stage indicate significant differences among treatments by LSD test (*P* < 0.05).

**FIGURE 4:**
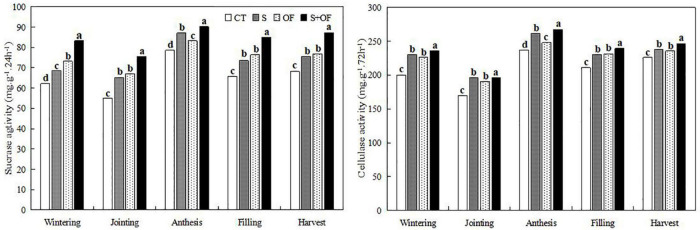
Effects of subsoiling, organic fertilizer and subsoiling combined with organic fertilizer on sucrase activity and cellulase activity of soil at different growth stages of winter wheat in 2019. CT, conventional tillage: S, subsoiling; OF, organic fertilizer; S+OF, organic fertilizer combined with subsoiling. Different lowercase letters in the same growth stage indicate significant differences among treatments by LSD test (*P* < 0.05).

**FIGURE 5:**
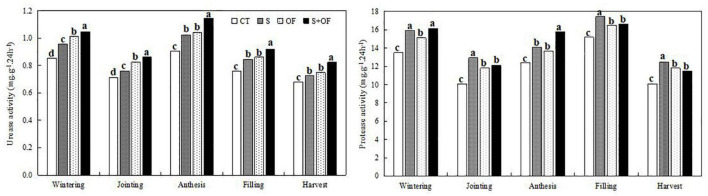
Effects of subsoiling, organic fertilizer and subsoiling combined with organic fertilizer on urease activity and protease activity of soil at different growth stages of winter wheat in 2019. CT, conventional tillage; S, subsoiling; OF, organic fertilizer; S+OF, organic fertilizer combined with subsoiling. Different lowercase letters in the same growth stage indicate significant differences among treatments by LSD test (*P* < 0.05).

**FIGURE 6:**
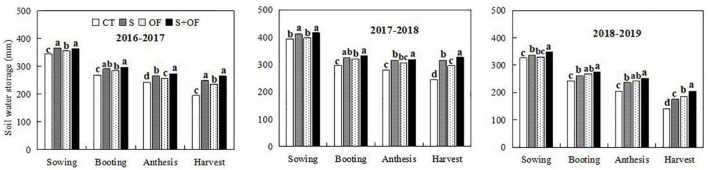
Effects of subsoiling, organic fertilizer and subsoiling combined with organic fertilizer on soil water storage (0–100 cm) at different growth stages of wheat in the three seasons of 2016–2019. CT, conventional tillage; S, subsoiling; OF, organic fertilizer; S+OF, organic fertilizer combined with subsoiling. Different lowercase letters in the same growth stage indicate significant differences among treatments by LSD test (*P* < 0.05).

**FIGURE 7:**
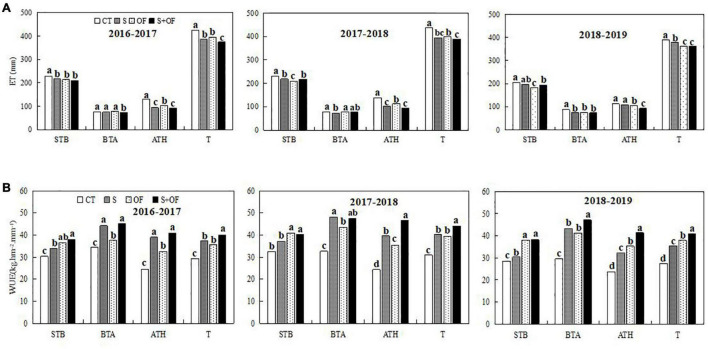
Effects of subsoiling, organic fertilizer and subsoiling combined with organic fertilizer on water consumption (ET) **(A)** and water use efficiency (WUE) **(B)** at different growth stages of winter wheat in the three seasons of 2016–2019. Note: STB, sowing to booting; BTA, booting to anthesis; ATH, anthesis to harvest; T(A), the total ET; T(B), the total WUE. CT, conventional tillage; S, subsoiling; OF, organic fertilizer; S+OF, organic fertilizer combined with subsoiling. Different lowercase letters in the same growth stage indicate significant differences among treatments by LSD test (*P* < 0.05).

The experimental data was statistically analyzed using SPSS 19.0. Three replicates were calculated for each treatment, and ANOVA was applied to compare whether different treatments were significantly different at *P* < 0.05 in Tables 1, 2 and Figures 2–7. The relationships between dry matter accumulation, soil water and soil organic carbon, soil microbial biomass carbon and nitrogen, and soil enzyme activity are described with the linear regression functions listed in Table 3. The relationships between yield of wheat and soil organic carbon, soil microbial biomass carbon and nitrogen, and soil enzyme activity are described with the linear regression functions listed in Table 4. The relationship between yield, WPE and dry matter accumulation, WUE of wheat at different growth stages under different treatments are described with the linear regression functions listed in Table 5.

**TABLE 1:** Effects of subsoiling, organic fertilizer and subsoiling combined with organic fertilizer on the dry matter accumulation (DM) at different growth stages of winter wheat in the three seasons of 2016–2019.

Year	Treatments	STJ	JTB	BTA	ATH	Total DM
2016–2017	CT	2487.0c	4170.4c	2640.0c	3175.7c	12473.0c
	S	2869.6b	4521.7b	3304.3a	3434.8b	14130.4b
	OF	3163.5a	4652.2ab	2977.4b	3256.5c	14049.7b
	S+OF	3269.6a	4700.0a	3269.6a	3555.7a	14794.8a
2017–2018	CT	2837.0c	4614.8c	2572.2c	3366.5c	13390.4c
	S	3104.3b	5021.7b	3515.2b	4063.0b	15704.3b
	OF	3287.0ab	5259.1a	3427.8b	3991.3b	15965.2b
	S+OF	3334.8a	5438.3a	3642.6a	4412.2a	16827.8a
2018–2019	CT	1944.3c	3888.7d	2580.0c	2654.8c	11067.8c
	S	2347.8b	4343.5c	3208.7ab	3482.6b	13382.6b
	OF	2415.2b	4523.0b	3087.1b	3675.5a	13700.9b
	S+OF	2556.5a	4884.8a	3332.6a	3834.8a	14608.7a

*STJ, sowing to jointing; JTB, jointing to booting; BTA, booting to anthesis; ATH, anthesis to harvest; Total DM, the total dry matter accumulation. CT, conventional tillage; S, subsoiling; OF, organic fertilizer; S+OF, organic fertilizer organic fertilizer combined with subsoiling. Different lowercase letters within a column mean significant difference between treatments at three seasons by LSD test (P < 0.05).*

**TABLE 2:** Differences in spike length, number of spikes, number of grains per ear, kilo-grain weightiness, yield and WPE of wheat between subsoiling, organic fertilizer and subsoiling combined with organic fertilizer in three seasons of 2016–2019.

Year	Treatments	Yield components				Yield (kg.hm^–2^)	WPE (kg.hm^–2^.mm^–1^)
		Spike length (cm)	Number of spikes (× 10^4^.hm^–2^)	Number of grains per ear	1,000-grain weightiness (g)		
2016–2017	CT	7.0c	439.1d	38.0c	43.5b	7025.8d	16.5c
	S	8.0a	608.7b	45.3a	43.2bc	7718.2b	20.0ab
	OF	7.7b	473.9c	42.4b	42.7c	7386.5c	18.7b
	S+OF	7.5b	625.2a	40.6b	44.7a	7841.4a	21.0a
2017–2018	CT	6.3c	520.4c	37.1c	43.1c	7578.3c	17.0c
	S	7.0a	640.9b	43.1a	46.6b	9087.4b	23.1ab
	OF	6.9ab	630.5b	41.3b	45.2b	9018.6b	22.5b
	S+OF	6.7b	700.0a	42.3ab	48.7a	9355.5a	24.1a
2018–2019	CT	6.8c	437.0c	31.5c	42.1c	6591.2c	16.3d
	S	7.3b	513.1b	36.5b	44.3ab	7593.2b	20.1c
	OF	7.4ab	530.4b	37.9ab	44.0b	7625.4b	21.0b
	S+OF	7.6a	600.0a	39.7a	45.9a	8475.8a	23.5a

*WPE, yield water production efficiency; CT, conventional tillage; S, subsoiling; OF, organic fertilizer; S+OF, organic fertilizer organic fertilizer combined with subsoiling. Different lowercase letters within a column mean significant difference between treatments at three seasons by LSD test (P < 0.05).*

**TABLE 3:** Relationships among dry matter accumulation, soil water storage and soil organic carbon, soil microbiomass carbon and nitrogen, and soil enzyme activity.

Index	Organic carbon	Soil microbial biomass carbon	Soil microbial biomass nitrogen	Sucrase activity	Cellulase activity	Urease activity	Protease activity
Dry matter accumulation	0.892**	0.858**	0.807**	0.894**	0.928**	0.780**	0.326
Soil water storage	0.384	0.698*	0.828**	0.542	0.475	0.884**	0.713**

*Means significant correlation at 0.05 level, and **means extremely significant correlation at 0.01 level.

**TABLE 4:** Relationships among yield of wheat and soil organic carbon, soil microbiomass carbon and nitrogen, and soil microorganisms enzyme activity.

Index	Wintering	Jointing	Anthesis	Filling
Soil organic carbon	0.969*	0.978*	0.960*	0.959*
Soil microbiomass carbon	0.963*	0.988*	0.987*	0.953*
Soil microbiomass nitrogen	0.977*	0.999**	0.992**	0.968*
Sucrase	0.968*	0.994**	0.948	0.989*
Cellulase	0.920	0.864	0.905	0.958*
Urease	0.943	0.928	0.995**	0.988*
Protease	0.898	0.695	0.988*	0.628

*Means significant correlation at 0.05 level, and **means extremely significant correlation at 0.01 level.

**TABLE 5:** Relationships among yield, WPE, dry matter accumulation, WUE of wheat at different growth stages under different treatments.

Treatments	Parameters	Yield			WPE		
		STB	BTA	ATH	STB	BTA	ATH
CT	DM	0.962**	–0.182	0.934**	0.234	0.275	0.299
	WUE	0.722*	0.516	0.214	0.129	0.280	0.278
S	DM	0.889**	0.916**	0.948**	0.727*	0.715*	0.882**
	WUE	0.868**	0.966**	0.822**	0.718*	0.881**	0.732*
OF	DM	0.745*	0.959**	0.877**	0.288	0.887**	0.943**
	WUE	0.887**	0.823**	0.557	0.810**	0.832**	0.790*
S+OF	DM	0.660	0.907**	0.969**	0.275	0.690*	0.813**
	WUE	0.819**	0.633	0.971**	0.655	0.624	0.842**

DM, Dry matter accumulation; WUE, water use efficiency; WPE, yield water production efficiency; STJ, sowing to jointing; JTB, jointing to booting; BTA, booting to anthesis; ATH, anthesis to harvest. CT, conventional tillage; S, subsoiling; OF, organic fertilizer; S+OF, organic fertilizer organic fertilizer combined with subsoiling. WPE, water production efficiency. *Means significant correlation at 0.05 level, and **means extremely significant correlation at 0.01 level.

## Results

### Effects of Combining Subsoiling and Organic Fertilizer on Physical and Chemical Properties of the Soil

#### Soil Structure

As shown in Table 6. Subsoiling (S), organic fertilizer (OF), and organic fertilizer combined with subsoiling (S+OF) increased the proportion of 0.5–1, 1–2, 2–3, 3–5, and >5 mm aggregates significantly. S+OF treatment showed a larger effect on >3 mm aggregates than S and OF treatments. However, the proportion of <0.25 mm aggregates under CT and S treatments was higher than that under OF and S+OF. S+OF resulted in the highest proportion of >0.25 mm aggregate compared to other treatments.

**TABLE 6:** The proportion of different diameters of aggregates under subsoiling, organic fertilizer and subsoiling combined with organic fertilizer.

Treatments	The proportion of different diameters of aggregates (%)							
	>5 mm	3–5 mm	2–3 mm	1–2 mm	0.5–1 mm	0.25–0.5 mm	<0.25 mm	>0.25 mm
CT	25.0bc	4.7c	3.5b	4.6c	7.5b	9.4c	43.2a	54.8d
S	26.2b	5.7b	4.2a	5.3b	6.7c	10.4b	42.5a	58.4c
OF	24.3c	5.8b	4.3a	7.0a	10.4a	12.9a	35.2b	64.8b
S+OF	34.4a	6.6a	4.4a	5.4b	7.7b	8.5d	33.1b	66.9a

*CT, conventional tillage; S, subsoiling; OF, organic fertilizer; S+OF, organic fertilizer organic fertilizer combined with subsoiling. Different lowercase letters within a column mean significant difference between treatments by LSD test (P < 0.05).*

#### Soil Organic Carbon

Figure 2 shows that across the growth period of wheat, soil organic carbon first decreased (wintering stage), then increased (anthesis stage) and finally decreased again (filling and harvest). S, OF and S+OF treatments increased soil organic carbon compared to CT at the different growth stages of wheat, and there was no difference between the S and OF treatments. The organic carbon content under S+OF treatment was the highest. This indicates that subsoiling coupled with organic fertilizer was more effective at increasing soil organic carbon compared to solely S or OF treatment.

#### Soil Microbial Biomass Carbon and Nitrogen

Figure 3 shows that across the growth period of wheat, the content of microbial carbon and nitrogen in the soil first decreased, then increased, and finally decreased again. Soil microbial biomass carbon and nitrogen at the jointing stage were lowest, and that at the anthesis stage was highest, compared to other growth stages. Compared to the other treatments, S+OF treatment was more beneficial for soil microbial biomass carbon and nitrogen.

#### Soil Enzyme Activity

From Figures 4, 5, S+OF treatment improved sucrase activity, cellulase activity and urease activity of wheat compared to the other treatments. However, protease activity of S treatment was the highest compared to other treatments throughout all growth stages of wheat except the anthesis stage.

### Effects of Combining Subsoiling and Organic Fertilizer on Soil Water Storage

As shown in Figure 6, it was found that in comparison with CT and OF, S and S+OF significantly increased soil water storage during the various growth periods of wheat during two experiment years (2016–2017, 2017–2018). Furthermore, water storage under S and S+OF treatments at the harvest stage increased by 27.2, 28.9, and 35.6%, 33.0% compared with CT in these 2 years, respectively. At the harvest stage, the soil water storage under OF and S+OF treatments increased by 32.1, 5.7, and 46.6%, 17.3% compared to CT and S treatments, respectively. In addition, during the 3-year experiment, S+OF treatment led to the highest soil water storage.

### Effects of Combining Subsoiling and Organic Fertilizer on Dry Matter Accumulation

As shown in Table 1, during the 3 years of experimentation, OF and S+OF treatment markedly improved the dry matter accumulation (DM) at the STJ (from sowing to jointing) and JTB (from jointing to booting) stages. Compared to CT, the DM in OF and S+OF in the three seasons increased by 15.9–27.2%, 17.5–31.5%, and 11.6–16.3%, 12.7–25.6%, respectively. In addition, DM under S+OF treatment was the largest, followed by S treatment at the BTA (from booting to anthesis) and ATH (from anthesis to harvest) stages in 2016–2018. However, DM under OF and S+OF treatment was significantly higher than that of other treatments at the ATH stage in 2018–2019. The maximum total DM occurred under S+OF treatment in all three seasons. S+OF was more beneficial in increasing DM during the different growth stages, and the total DM of wheat.

### Effects of Combining Subsoiling and Organic Fertilizer on Water Consumption and Water Use Efficiency

As shown in Figures 7A,B, OF and S+OF treatments significantly reduced water consumption (ET) and increased water use efficiency (WUE) during STB (from sowing to booting). Compared with CT, WUE in OF and S+OF treatments was larger by 11.5–19.0% and 24.0–25.1%, respectively. At the BTA and ATH stages, S+OF treatment has the lowest ET and the highest WUE, followed by S treatment, except for the ATH stage in 2018–2019. However, ET under OF treatment was significantly lower than that under S and CT treatments at the ATH stage in 2018–2019, and WUE under OF treatment increased by 47.6 and 19.6% compared to S and CT, respectively. Moreover, S+OF treatment reduced the total ET[T(a)], while the total WUE [T(b)] under S+OF was significantly higher than other treatments during the whole growth period of wheat. The present study shows that S+OF treatment improves water storage and moisture retention, reduces ET, and increases the WUE of wheat. Therefore, the combination of S+OF led to greatly increased WUE.

### Effects of Combining Subsoiling and Organic Fertilizer on Wheat Yield and Yield Water Production Efficiency

Our results (Table 2) show that compared to CT, the spike length and the number of grains per ear under S treatment during 2016–2017 and 2017–2018 increased by 14.9, 19.1, and 8.2%, 16.1%, respectively, while those under S+OF treatment during 2018–2019 increased by 13.1 and 26.0%, respectively. Additionally, the number of spikes and the 1,000-grain weightiness under S+OF treatment were significantly higher than under other treatments. Compared with CT, the number of spikes and the 1,000-grain weightiness under S+OF treatment in all three seasons larger by 30.7–48.5% and 2.6–13.0%, respectively. The wheat yield and yield WPE of S+OF treatment were the highest in all three seasons, which was 11.6–28.6% and 26.8–43.6% larger compared to CT. In addition, the yield and WPE under S treatment were higher than those under OF and CT treatment in 2016–2018, while the yield and WPE under OF treatment were higher than those under S and CT treatment in 2018–2019.

### Correlation Analysis of Dry Matter Accumulation, Soil Water Storage and Soil Organic Carbon, Soil Microbial Biomass Carbon and Nitrogen, and Soil Enzyme Activity

From Table 3, dry matter accumulation showed significant (*P* < 0.05) or extremely significant (*P* < 0.01) positive correlations with organic carbon content, soil microbial biomass carbon and nitrogen, sucrase, cellulase, urease and protease activity. Soil water storage showed significant (*P* < 0.05) or extremely significant (*P* < 0.01) positive correlations with soil microbial biomass carbon and nitrogen, urease and protease activity.

### Correlation Analysis of Wheat Yield and Soil Organic Carbon, Soil Microbial Biomass Carbon and Nitrogen, and Soil Enzyme Activity

Soil organic carbon, soil microbial biomass carbon and nitrogen, sucrase, cellulase, urease, protease at different growth stages had positive correlations with the yield and WUE of wheat (Table 4). From Table 4, organic carbon content, soil microbial biomass carbon nitrogen, and sucrase at different stages of wheat showed significant (*P* < 0.05) or extremely significant (*P* < 0.01) positive correlations with yields. Cellulase and urease during the filling and harvest stages showed significant (*P* < 0.05) or extremely significant (*P* < 0.01) positive correlations with yield. Urease and protease at anthesis stage showed significant (*P* < 0.05) or extremely significant (*P* < 0.01) positive correlations with yield.

### Correlation Analysis of Dry Matter Accumulation, Water Use Efficiency at Different Growth Stages and Yield, Water Production Efficiency of Wheat at Different Growth Stages

Water use efficiency (WUE) and dry matter accumulation (DM) were correlated with wheat yield and WPE (Table 5). At the STB stage, DM and WUE in the all treatments were significantly (*P* < 0.05) or extremely significantly (*P* < 0.01) positively correlated to yields, except between DM and yield under S+OF treatment. DM of S, OF, and S+OF was extremely significantly (*P* < 0.01) positively correlated with the yield, and the WUE of the S and OF treatments were extremely significantly (*P* < 0.01) and positively correlated with the yield at the BTA stage. At the ATH stage, DM in all treatments were extremely significantly (*P* < 0.01) and positively correlated with yield, and the regression coefficient of S+OF treatment was higher (*R*^2^ = 0.969). In addition, the correlation between WUE and yield under S and S+OF treatments were also significant (*P* < 0.01) and positive. Moreover, DM and WUE under S treatment were significantly (*P* < 0.05) and positively correlated with WPE at the STB stage. At the BTA stage, DM and WUE under S and OF treatments were significantly (*P* < 0.05) or extremely significantly (*P* < 0.01) and positively correlated with WPE. At the ATH stage, DM and WUE under S, OF and S+OF treatments were significantly (*P* < 0.05) or extremely significantly (*P* < 0.01) and positively correlated with WPE.

## Discussion

### Effects of Combining Subsoiling and Organic Fertilizer on Soil Structure, Microbial Biomass Carbon and Nitrogen and Soil Enzymes

Subsoiling, organic fertilizer, and the combination of these two practices improved soil aggregate structure, microbial biomass carbon and nitrogen and soil enzymes. We found that S, OF and S+OF treatments increased the proportion of >0.5 mm aggregates significantly. Among these, S+OF treatment was most beneficial for increasing the proportion of >0.25 mm aggregates because of the synergistic effects of the combination of S+OF: subsoiling provides loose soil conditions (Yang et al., 2021b) and leads to increased water availability in the root zone, which promotes crop root growth and increases soil organic carbon (Yang et al., 2021b), while organic fertilizers provide nutrients to the crop and soil microorganisms (Spedding et al., 2004; Wang et al., 2015) and thereby promote microbial activity (Piovanelli et al., 2006; Kuzyakov and Xu, 2013) and increase soil organic carbon (Yang et al., 2021a). These factors in turn increased soil structure stability, increased soil water retention, and improved crop growth. The combination of S+OF this results in a positive feedback loop of crop growth and improvements in soil physical properties.

Microbial biomass carbon and nitrogen in the soil can directly or indirectly participate in soil biochemical processes, and plays an extremely important role in the transformation of substances and the energy cycle in the soil (Turner et al., 2001; Nsabimana et al., 2004). In this study, we found that throughout the process of wheat growth, changes in the content of soil microbial biomass carbon and nitrogen had the same trend as soil organic carbon. Soil microbial biomass carbon at the jointing stage and soil microbial nitrogen at the jointing and harvest stages were lowest, and that at the anthesis stage was highest, compared to other growth stages. Previous research has shown that the application of organic fertilizer can effectively increase the carbon content of soil microbes during the crop growth stage, with a 23.0% increase in average annual content (Li et al., 2017). We found that compared to CT, S, and OF treatments, S+OF treatment led to more soil microbial biomass carbon and nitrogen. This indicates that compared to individual application of subsoiling tillage or organic fertilizer, their combination better improves the physical and chemical characteristics of the soil, increases root exudates, promotes nutrient absorption by crop roots, and enhances soil microbial activity and reproduction (Yu, 2015), and microbial biomass quantity (Kuzyakov and Xu, 2013).

Soil enzymes are good catalysts for nutrient metabolism in the soil. Enzyme activity can reflect the extent of decomposition and transformation of substances in the soil, and also reflect changes to soil fertility due to farmland management measures (Zhang et al., 2014). Studies have shown that subsoiling can significantly improve the activity of urease and sucrase in the soil (Huang et al., 2013), while organic fertilizer improves the activity of urease, sucrase, and cellulase in the soil (He et al., 2020). We found that S+OF improved sucrase activity, cellulase activity and urease activity of wheat to a greater extent, compared to sole application of either subsoiling or organic fertilizer. However, protease activity was the highest under S treatment throughout all growth stages of wheat except the anthesis stage. Subsoiling, which creates small disturbances to the soil, increases soil porosity, promotes gas exchange, increases water retention, and facilitates enzyme activity in the soil (Jiang et al., 2012; Nie et al., 2015). Organic fertilizers can increase the carbon content of the soil, and increase root exudates and microbial activity, which increases enzyme activity in the soil (Rehana et al., 2008; Pu et al., 2020). Therefore, the combination of subsoiling combined with organic fertilizer (S+OF) is a good practice to improve the activity of urease, sucrase, and cellulase in the soil and promote crop growth.

### Effects of Combining Subsoiling and Organic Fertilizer on Soil Water Storage and Dry Matter Accumulation

Soil water retention is a critical factor and important parameter for evaluating soil productivity, as it has a decisive influence on crop growth conditions and yield (Fu et al., 2005; Yang et al., 2021c). Subsoiling and organic fertilizer can improve soil structure, and promote water storage and infiltration to deeper soil (Hemmat and Eskandari, 2004; Wang et al., 2019; Hu et al., 2013; Liu et al., 2013). In this study, it was found that in comparison with CT and OF, S and S+OF significantly increased soil water storage during the various growth periods of wheat during two experimental years (2016–2017, 2017–2018). This may be due to the better infiltration and soil water retention capacities induced by subsoiling (Hu et al., 2013). Compared to CT and S treatments, OF and S+OF treatments were more effective at increasing soil water storage in 2018–2019, because organic fertilizers improve the soil water retention capacity and moisture content by improving the organic matter content and soil physical properties (Karami et al., 2012). Thus, the application of organic fertilizer leads to higher soil water storage when comparing treatments subject to the same tillage process in the dry growth season. In addition, during the 3-year experiment, S+OF treatment led to the highest soil water storage during the growth stage of wheat, which may be because the synergies between subsoiling and organic fertilizer significantly improved soil and water conservation capacity (Bolan et al., 2003). Therefore, S+OF treatment appears to be most effective at increasing soil water retention capacity.

The accumulation of dry matter during the growth period provides the fundamental materials required for crop formation, which ultimately determines grain yield (Ding et al., 2005; Liu et al., 2009; Hou et al., 2012). Different tillage and cultivation measures can improve the dry matter accumulation ability of wheat by improving soil moisture (Zheng et al., 2013). We found that, during the 3 years of experimentation, OF and S+OF treatment markedly improved dry matter accumulation (DM) at the STJ (from sowing to jointing) and JTB (from jointing to booting) stages. Our results show that the input of organic fertilizer increased DM before the booting stage, which agrees with Yang et al. (2016). This is because organic fertilizer leads to more balanced crop water demand and soil water supply (Liu et al., 2013), improves the effective water content, promotes the transformation of soil organic nutrients, and improves the absorption and utilization of crop nutrients and water, thus promoting the growth of wheat and increasing DM (Dordas, 2009). In addition, our results also show that the application of organic fertilizer may increase post-anthesis DM during the dry growth season (2018–2019). The reason for this may be that organic fertilizer enhanced deep soil water usage in arid years, which promotes ear development and grain filling (Lu et al., 2011; Ni et al., 2013; Wang et al., 2017), thereby resulting in increased DM. However, among different practices, application of organic fertilizer coupled with subsoiling (S+OF) was more beneficial in increasing DM than sole subsoiling due to double advantage for soil microbes, soil properties and water retention mentioned above.

### Effects of Combining Subsoiling and Organic Fertilizer on Water Consumption, Water Use Efficiency, and Water Production Efficiency of Wheat

Miriti et al. (2012) reported that when subject to identical tillage practices, the water use efficiency under organic fertilizer treatment was 26% higher than without organic fertilizer. In this study, OF and S+OF treatments significantly reduced ET and increased WUE during STB (from sowing to booting). These results agree with several previous studies (Lu et al., 2011; Miriti et al., 2012; Zhang et al., 2016; Wang et al., 2017). This may because organic fertilizers help conserve water and soil moisture, reduce inter-plant evaporation and promote the absorption and utilization of water by roots (Lu et al., 2011). In addition, we found that OF treatment led to reduced ET and increased WUE during ATH compared to S treatment during the dry growth season, thus increasing wheat yield. Moreover, S+OF treatment reduced the total ET, while the total WUE under S+OF treatment was significantly higher than other treatments. The present study shows that S+OF treatment improves water storage and moisture retention, reduces ET, and increases the WUE of wheat. This is due to an improvement in infiltration, reduction in surface runoff and soil erosion, and increase in WUE due to subsoiling (Mcconkey et al., 1997; Hu et al., 2013). Furthermore, organic fertilizer could also increase WUE (Miriti et al., 2012; Zhang et al., 2016). Therefore, the combination of S+OF led to greatly increased WUE.

The increased soil water storage, post-anthesis DM and the number of spikes and 1,000-grain weight caused by subsoiling (Huang et al., 2009; Zheng et al., 2011; Yang et al., 2016), and the benefits of organic fertilizer in improving soil physical properties, increasing soil water storage capacity, reducing evaporation, and improving crop yields (Sang et al., 2016) and the absorption and utilization of water (Lu et al., 2011; Miriti et al., 2012; Liu et al., 2013; Wang et al., 2017), suggests that the two individual practices in S+OF provide mutually complementary benefits to wheat growth. Therefore, compared to S and OF treatments, S+OF treatment optimized yield components (number of spikes and 1,000-grain weightiness), which led to a higher number of spikes, 1,000-grain weightiness and total DM and lower ET, and increased WPE and wheat yield.

Correlation analyses indicate that high dry matter accumulation and soil water improved soil microbial biomass carbon and nitrogen and soil enzyme activity, which improves soil organic carbon and soil structure and promotes crop growth. We found that the increment in soil microbial biomass carbon and nitrogen at the different growth stages of winter wheat, and the increment in soil sucrase, cellulase, urease and protease activity after anthesis stage of winter wheat were beneficial to increase yield and WPE of winter wheat based on the correlation analysis. In addition, we also found that dry matter accumulation at the different growth stages of winter wheat had a critical and positive impact on yields and WPE under S, OF and S+OF treatments, especially after the anthesis stage under S+OF treatment (Table 5), and that improvements in WUE after the booting stage under S, OF and S+OF treatments, especially after the anthesis stage under S+OF treatment (Table 5), led to increased wheat yield and WPE, in agreement with previous studies (Zheng et al., 2008; Yang et al., 2016). Zheng et al. (2008) reported that the dry matter accumulation of winter wheat after anthesis accounted for more than 80% of total grain yield. This is also reflected in our results, which show that S+OF treatment was highly effective at increasing DM and WUE post-anthesis, thereby increasing yield and WPE. Thus, appropriate agricultural practices such as S+OF can effectively improve soil organic carbon, promote soil microbial biomass carbon and nitrogen and soil enzyme activity, thereby increasing wheat production.

## Conclusion

Subsoiling (S), organic fertilizer (OF) and subsoiling combined with organic fertilizer (S+OF) are treatments that improve soil structure. Among them, S+OF is most beneficial in increasing the proportion of >0.25 mm aggregates, which leads to higher soil structural stability. In addition, S+OF is also the most effective measure in increasing soil organic carbon content, soil microbial biomass carbon, soil microbial biomass nitrogen and sucrase activity, cellulase activity, urease activity and protease activity. Furthermore, S+OF also improved the absorption and utilization of water and DM, wheat yields, and WPE, more significantly compared to other treatments, regardless of rainfall availability. Correlation analyses indicate that long-term continuous amendments are beneficial for improving wheat yield and WPE because they improve soil organic carbon, soil microbial biomass carbon and nitrogen, soil enzyme activity, dry matter accumulation and soil water storage. Therefore, subsoiling combined with organic fertilizer is an effective measure for improving the soil and increasing yield and WPE of winter wheat production under the experimental conditions. Further research is necessary to determine the effects of long-term application of S+OF on the mechanisms of the interactions between microbial variety, and microbial quantity, soil properties, and water availability, so as to build a more informed scientific basis for applying S+OF in farmland.

## Data Availability Statement

The raw data supporting the conclusions of this article will be made available by the authors, without undue reservation.

## Author Contributions

YY wrote the main manuscript. ML wrote the part of the manuscript. JW revised and gave some advice for the manuscript. XP performed most of the experiments. CG prepared the figure and table of the manuscript. DT edited language and modified the main manuscript. All authors reviewed the manuscript.

## Conflict of Interest

The authors declare that the research was conducted in the absence of any commercial or financial relationships that could be construed as a potential conflict of interest.

## Publisher’s Note

All claims expressed in this article are solely those of the authors and do not necessarily represent those of their affiliated organizations, or those of the publisher, the editors and the reviewers. Any product that may be evaluated in this article, or claim that may be made by its manufacturer, is not guaranteed or endorsed by the publisher.
